# Supplementary material for: Analysis of local and systemic side effects of bacillus Calmette-Guérin immunotherapy in bladder cancer: a retrospective study in Türkiye
Source: PeerJ. 2025 Feb 12;13:e18870. doi: 10.7717/peerj.18870 (PMC11829628; doi:10.7717/peerj.18870)
Supplement: Supplemental Information 2 [file peerj-13-18870-s002.docx]

RAW DATA CODEBOOK

1. For the conceptual data stated below; 0: absent 1: present.

Comorbidities, Diabetes mellitus, Hypertension, Benign prostatic hyperplasia, Any side effects, Minor side effects, Fever, Cystitis, Hematuria, Dysuria, Major side effects, Sepsis, Septic shock, Arthritis, Pneumonia, Lymphadenopathy, Hepatitis

1. If there is growth in the "Urine culture" category, it is indicated in the table. 0 means no growth.
2. In the category "Reason for terminating treatment", "0" is written if there is no treatment termination for any reason

Herhangi bir komplikasyon: Any side effects

Minor komplikasyon (ateş, sistit, hematüri, dizüri): Minor side effects (Fever, Cystitis, Hematuria, Dysuria)

ateş: Fever

sistit: Cystitis

hematüri: Hematuria

dizüri: Dysuria

Major komplikasyon (sepsis, septik sok,artrit, pnomoni,LAP, HEPATİT) : Major side effects ( Sepsis, Septic shock, Arthritis, Pneumonia, Lymphadenopathy, Hepatitis)

septik şok: Septic shock

artrit: Arthritis

pnömoni:   Pneumonia

LAP: Lymphadenopathy

HEPATİT: Hepatitis

kaç doz BCG: Number of BCG therapies

KAÇINCIDA SEMPTOM: Symptom on which therapy

YIL: Year of BCG therapy

idrar kültürü: Urine culture

TEDAVİ SONLANDIRMA NEDENİ: Reason for terminating treatment
